# The association of cumulative low-density lipoprotein cholesterol exposure and carotid intima-media thickness in a young adulthood population

**DOI:** 10.1186/s12872-024-03977-x

**Published:** 2024-06-24

**Authors:** Wei Huang, Weiqi Nie, Jianqiu Zhao, Zhihui Fan, Dan Wang, Xia Wu, Yujing Hao, Shouling Wu

**Affiliations:** 1 Department of Ultrasound, Kai Luan General Hospital, Tangshan, China; 2 Department of Ultrasound, Tangshan People’s Hospital, Tangshan, China; 3 Department of Anesthesiology, Pudong Medical Center, Shanghai Pudong Hospital, Fudan University, Shanghai, China; 4 Department of Cardiology, KaiLuan General Hospital, Tangshan, China

**Keywords:** Low density lipoprotein cholesterol cumulative exposure, Time-weighted average, Carotid diamantine thickness, Young adulthood population, Atherosclerotic cardiovascular disease

## Abstract

**Objective:**

To investigate the association between cumulative exposure to low-density lipoprotein cholesterol (LDL-C) and carotid intima-media thickness (IMT) in the young adulthood population.

**Methods:**

Young adult subject (18-45 year old) from the Kailuan Study group who participated in the same period of follow-up and received carotid artery ultrasound were selected as the observation subjects. Among them, 3651 cases met the inclusion criteria, which required that carotid artery color ultrasound examinations be completed from 2010 to 2016, with complete IMT measurements, LDL-C data collected at least twice before carotid ultrasound, and participants’ age to be ≤ 45 years at the time of carotid artery color ultrasound examination. Linear regression was used to analyze the correlation between time-weighted average (TWA) to LDL-C cumulative exposure and IMT the young population. Logistic regression was used to analyze the effects of different TWA groups on IMT thickening. Considering that the use of anti hypertensive drugs and lipid-lowering drugs may affect TWA LDL-C, this study excluded people taking antihypertensive drugs and lipid-lowering drugs, and conducted a repeat analysis of the main results.

**Results:**

There was a positive correlation between TWA LDL-C and IMT, with IMT increasing by 0.017 mm when TWA LDL-C increased by 1 mmol/L * year. The TWA LDL-C in the highest group was identified as a risk factor for IMT thickening, with odds ratio (*OR*) values of 1.812(1.027 ~ 3.200) in the T3 group. After excluding patients taking antihypertensive drugs and lipid-lowering drugs, the results still showed that the T3 group with the highest TWA LDL-C was a risk factor for IMT thickening, with an *OR* value of 1.850(0.988–3.464), *P* for trend is 0.043.

**Conclusion:**

This cohort study revealed that TWA LDL-C is positively correlated with IMT in young adulthood for risk stratification, and control LDL-C levels at an earlier age may reduce the lifetime risk of developing atherosclerotic disease.

**Trial registration:**

ChiCTR-TNC-11001489.

## Background

Genetics, epidemiology, and randomized controlled trials (RCT) all confirm that dysfunction of lipid metabolism, represented by low density lipoprotein cholesterol (LDL-C), is a risk factor for atherosclerotic cardiovascular disease (ASCVD) [1]. Current lipid management guidelines are primarily intended for individuals aged 40–70 years, and there is a lack of treatment recommendations for young people at low risk of cardiovascular disease [2]. This is primarily due to several factors. Firstly, the low incidence of cardiovascular events in young populations makes it challenging to assess the impact of lipid metabolism disorders on ASCVD. Secondly, longer observation and follow-up periods are needed to observe these effects. Lastly, conducting RCT in this demographic presents logistical challenges. Clinical atherosclerosis, such as thickening of the inner media of the artery, can be used as an alternative endpoint.

Although there are many studies on the relationship between LDL-C and ASCVD, there is a lack of studies on the relationship between LDL-C and diamantine thickness (IMT) in young people. Additionally, the only observational population is mostly European and American people [3, 4], while data on Asian people are lacking. Furthermore, previous studies on the association between LDL-C and carotid IMT in young adults mostly used single LDL-C data [5, 6], while recent studies have found that cumulative exposure to risk factors has a greater impact on adverse outcomes than single measurements [7–9]. Most observational studies on the association between LDL-C and ASCVD have focused on LDL-C level at a single time point (usually in middle or older age), and few studies have characterized long-term exposures to LDL-C and their role in ASCVD risk. To explore the effects of LDL-C on carotid diamantine thickening in young people, we analyzed the association between cumulative exposure time-weighted average (TWA) to LDL-C and carotid diamantine thickness in a young adulthood population based on the data from the cohort of Kailua study (registration number ChiCTR-TNC-11001489).

## Methods

### Study design and population

The observation subjects were from the Kailua study cohort. The Kailua study began in 2006 and was followed up every 2 years to detect blood lipid indexes, including LDL-C. Carotid artery ultrasonically was performed on part of the subjects at the 2nd to 5th follow-up using a non-random method. We studied a cohort of young adults who participated in concurrent follow-up and underwent carotid ultrasound. This study was approved by the ethics committee of Kailua Medical Group ([2006] Approval No.5). Informed consent is signed by all study participants.

**Fig. 1:**
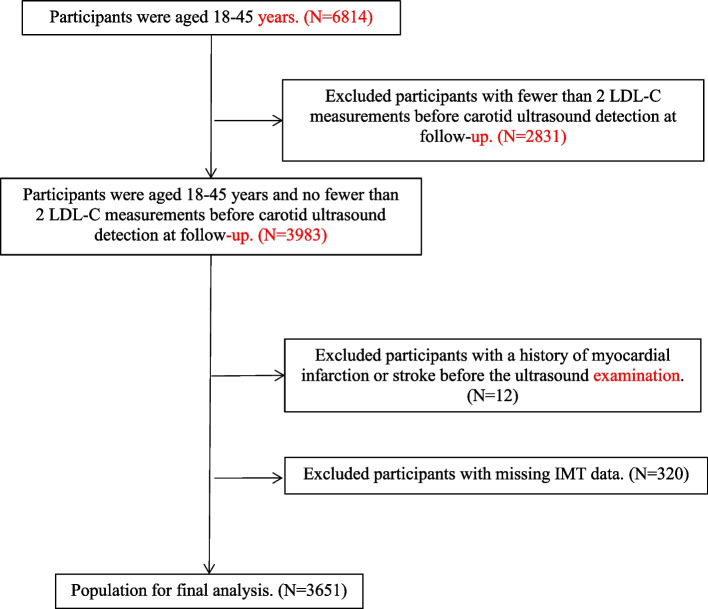
Flowchart of the study participants

A total of 23,200 participants participated in the Kailua study follow-up from 2006 to 2016 and underwent carotid ultrasound during the follow-up from 2010 to 2016. Among them, 6 814 were aged 18–45 years, and 2 831 with fewer than 2 LDL-C measurements before carotid ultrasound were excluded. Additionally, 12 patients with a history of myocardial infarction or stroke before the ultrasound examination and 320 patients with missing IMT data were excluded. Finally, 3 651 cases were included in the statistical analysis (Fig. 1).

### Data collection

The epidemiological investigation contents, anthropometric indicators, and biochemical indicators can be found in the previous published literature of this research group [10].

### Measurement of IMT

On the day of the physical examination, carotid artery ultrasound examinations were conducted by ultrasound doctors who had been engaged in ultrasound work for more than 5 years and had been uniformly trained. A PHILIPS HD-15 color ultrasonic diagnostic instrument was used, and the probe frequency was 5 ~ 12MHZ. The subjects were in a supine position, and their heads was tilted to the examination side to side. Bilateral common carotid arteries were routinely scanned and IMT was measured at a location 1 ~ 2 cm near the heart of the common carotid artery bifurcation. The highest values of bilateral carotid IMT measurements were recorded. Result evaluations: Two sonographers with more than 5 years of working experience jointly reviewed and confirmed the ultrasound images based on the criteria mentioned above. Different sonographers randomly re-examined carotid artery ultrasound and re-evaluated the IMT results, and the retest reliability was 97%. The highest value of bilateral carotid artery IMT was used. The carotid artery color Doppler ultrasound data from the same period of physical examination were selected as baseline data, and the LDL-C measured at that time was considered the baseline LDL-C. We defined IMT > 0.9 mm as the IMT thickening group and IMT ≤ 0.9 mm as the IMT normal group [11].

### Function definition and properties

The cumulative exposure time was calculated as the time between the first LDL-C measurement and the actual participation in color Doppler ultrasound. The mixed-effects model was used to estimate the cumulative LDL-C exposure value. The time-weighted cumulative LDL-C (cum LDL-C) was defined as the product of the mean total LDL-C scores of two tests (baseline and follow-up tests) multiplied by years of consecutive follow-up: cum LDL-C = (LDL-C1 + LDL-C2) /2 × time1-2 + (LDL-C2 + LDL-C3) /2 × time 2–3 + LDL-C3 + LDL-C4) /2 × time 3–4 + (LDL-C4 + LDL-C5) /2 × time 4–5. LDL-C1, LDL-C2, LDL-C3, LDL-C4 and LDL-C5 refer to the LDL-C values of the study subjects who participated in the 1st, 2nd, 3rd, 4th and 5th physical examinations, respectively. Time1-2, time 2–3, time 3–4, and time 4–5 refer to the time interval for the subjects to participate in the health examination for 1 to 5 consecutive times. And those less than 5 times are calculated according to the actual number. The TWA LDL-C was calculated as the cumulative LDL-C exposure value divided by the cumulative exposure time. The participants were stratified by tertiles of TWA LDL-C: T1 group, TWA < 2.35 mmol/L * year (1212cases), T2 group, 2.35 mmol/L * year ≤ TWA < 2.63 mmol/L * year (1243 cases), and T3 group, TWA > 2.63 mmol/L * year(1196 cases).

Smoking is defined as smoking at least one cigarette per day for a continuous period of at least one year or quitting for less than one year. Alcohol consumption was defined as consuming ≥ 2 standard drinks per day (with the WHO standard drink being 10 g of pure alcohol) for a duration of ≥ 1 year. Hypertension is defined as systolic blood pressure ≥ 140 mmHg or diastolic blood pressure ≥ 90 mmHg or a history of high blood pressure or taking blood pressure medications. Diabetes was defined as fasting blood glucose > 7.0 mmol/L or a history of diabetes or taking hypoglycemic drugs. Highly sensitive C-reactive protein (Hs-CRP) levels were divided into three groups: low level (Hs-CRP < 1.0 mg/L), medium level (1.0 mg/L ≤ Hs-CRP ≤ 3.0 mg/L), and high level (Hs-CRP > 3.0 mg/L). Body mass index (BMI) was classified as normal (BMI < 24 kg/m2), overweight (24 kg/m2 < BMI < 28 kg/m2), and obese (BMI > 28 kg/m2).

### Statistical approach

Measurement data with a normal distribution were expressed as mean ± standard deviation. Single factor analysis of variance or rank sum test were used for comparison between groups. Statistical data were presented as rate or component ratio, and the *χ*^*2*^ test was used for comparison between groups. Linear regression was used to analyze the correlation between LDL-C cumulative exposure time weighted average and IMT in individuals 18–45 years of age, with *P* < 0.05 considered statistically significant. Logistic regression was used to analyze the effects of different TWA groups on IMT thickening in people 18–45 years old, with *P* < 0.05 considered statistically significant. Sensitivity analysis: Considering that the use of antihypertensive and lipid-lowering drugs may affect the weighted average cumulative exposure time of LDL-C, this study did a repeat analysis of the main results after excluding the population taking antihypertensive and lipid-lowering drugs.

Statistical analysis was performed using SAS 9.4 and SPSS 22.0 statistical software.

## Result

### Characteristics of participants according to tripartite of TWA LDL-C

Among the 3 651 subjects included in the analysis, the mean age was 37 ± 5 years, the mean number of LDL-C measurements was 4 ± 1 times, and the cumulative LDL-C exposure time was 6.3 ± 2.6 years. From T1 to T3, the proportion of men, smokers, obese individuals, individuals with high Hs-CRP levels, hypertension, diabetes, and those taking antihypertensive drugs, as well as the levels of triglyceride and IMT gradually increased. Conversely, the proportion of individuals with normal BMI and high density lipoprotein (HDL-C) levels gradually decrease. The age of T3 group was higher than that of T1 and T2 groups. The proportion of university degree or above in T3 group was lower than that in T1 and T2 group. The proportion of alcohol consumption in the T1 group was higher than that in the other two groups. The differences between the above groups were statistically significant (Table 1).

**Table 1:** Characteristics of participants according to tripartite of time-weighted cumulative LDL-C

Characteristics	Total	T1 (< 2.35)	T2 (2.35 to 2.63)	T3 (> 2.63)	*P*-value
Number of participants	3651	1212	1243	1196	
Male, n (%)	1931 (52.9)	477 (39.3)	706 (57.5)	748 (61.9)	< 0.001
Age, years	36.5 ± 5.5	36.4 ± 5.5	36.1 ± 5.7	36.9 ± 5.4	0.002
University degree or above, n (%)	1475 (40.7)	507 (42.1)	521 (42.7)	447 (37.3)	0.012
Smoke, n (%)	1091 (30.0)	253 (20.9)	407 (33.2)	431 (35.7)	< 0.001
Drink n (%)	1731 (47.4)	609 (50.2)	540 (43.9)	582 (48.1)	< 0.001
BMI grouping, n (%)					
< 24.0 kg/m^2^	1705 (47.4)	709 (59.6)	581 (48.1)	415 (34.7)	< 0.001
≥ 24.0 kg/m^2^, < 28.0 kg/m^2^	1271 (35.3)	346 (29.1)	410 (33.9)	515 (43.1)	
≥ 28.0 kg/m^2^	619 (17.3)	134 (11.3)	219 (18.1)	266 (22.2)	
Hs-CRP grouping, n (%)					
< 1.0 mg/L	2023 (59.0)	644 (57.7)	753 (64.6)	626 (54.4)	< 0.001
≥ 1 mg/L, ≤ 3 mg/L	927 (27.0)	329 (29.5)	251 (21.6)	347 (30.2)	
> 3 mg/L	481 (14.0)	143 (12.8)	161 (13.8)	177 (15.4)	
HDL-C, mmol/L	1.44 ± 0.54	1.51 ± 0.49	1.39 ± 0.37	1.42 ± 0.71	< 0.001
Triglyceride, mmol/L	1.52 ± 1.26	1.33 ± 1.25	1.49 ± 1.17	1.74 ± 1.32	< 0.001
Hypertension, n (%)	588 (17.5)	148 (14.0)	195 (17.1)	245 (21.4)	< 0.001
Diabetes, n (%)	115 (3.2)	24 (2.06)	32 (2.67)	59 (5.00)	< 0.001
Take blood pressure medication, n (%)	193 (5.3)	57 (4.7)	56 (4.6)	80 (6.6)	0.040
Take lipid-lowering drugs, n (%)	32 (0.8)	6 (0.49)	10 (0.81)	16 (1.32)	0.088

*Abbreviations*: *LDL-C *Low density lipoprotein cholesterol, *T1 ~ T3* Divided into three groups according to the weighted mean cumulative exposure time of low density lipoprotein cholesterol, *BMI* Body mass index, *Hs-CRP* Highly sensitive C-reactive protein, *HDL-C* High-density lipoprotein cholesterol

Continuous variables are expressed as mean ± SD. Categorical variables are expressed as frequency (percentage)

### Carotid IMT of participants according to tripartite of TWA LDL-C

The average carotid IMT in the observation population was 0.68 ± 0.15 mm, among which 118 cases (3.2%) exhibited thickening. Both IMT thickness and the proportion of IMT thickening population showed an increasing trend, and the differences between groups were statistically significant (Table 2).

**Table 2:** IMT date of participants according to tripartite of time-weighted cumulative LDL-C

Characteristics	Total	T1 (< 2.35)	T2 (2.35 to 2.63)	T3 (> 2.63)	*P*-value
IMT (mm)	0.68 ± 0.15	0.66 ± 0.14	0.68 ± 0.14	0.71 ± 0.15	< 0.001
IMT thicken n (%)	118 (3.2)	23 (1.9)	39 (3.2)	56 (4.6)	< 0.001

*IMT* Carotid intima-media thickness, *LDL-C* Low density lipoprotein cholesterol, *T1 ~ T3 *Divided into three groups according to the weighted mean cumulative exposure time of low density lipoprotein cholesterol

### Linear regression analysis of TWA LDL-C and IMT

Linear regression was used to analyze the correlation between TWA LDL-C and IMT in individuals 18–45 years of age. IMT was used as the dependent variable and TWA as the independent variable in the linear regression model. Model 1 involved a single factor analysis, and model 2 corrected for age and sex. Model 3 was adjusted for education level, body mass index, smoking, alcohol consumption, diabetes, hypertension, HDL-C, triglyceride and Hs-CRP on the basis of model 2. Model 4 added adjustments for the use of antihypertensive drugs and lipid-lowering drugs to model 3, the adjustment of was added. After adjusting for possible influencing factors, the results showed that TWA LDL-C was positively correlated with IMT, with IMT increasing by 0.017 mm for every 1 mmol/L year increase in TWA LDL-C (*P* < 0.05) (Table 3).

**Table 3:** Linear regression analysis of weighted average cumulative exposure time of LDL-C and IMT

	T-value	*β*-value	*P*-value	95%*CI*
Model 1				
TWA LDL-C	9.4	0.059	< 0.001	0.047 ~ 0.071
Model 2				
TWA LDL-C	3.65	0.021	< 0.001	0.010 ~ 0.033
Model 3				
TWA LDL-C	2.60	0.017	0.009	0.004 ~ 0.029
Model 4				
TWA LDL-C	2.62	0.017	0.009	0.004 ~ 0.029

Model 1: Single factor linear regression analysis was performed with IMT as the dependent variable and TWA LDL-C as the independent variable. Model 2: Age and sex were corrected based on model 1. Model 3: On the basis of model 2, adjustments were added for education level, body mass index, smoking, alcohol consumption, diabetes, hypertension, HDL cholesterol, triglycerides, and C-reactive protein. Model 4: On the basis of model 3, the adjustment of taking antihypertensive drugs and lipid-lowering drugs was added

*LDL-C *Low density lipoprotein cholesterol, *IMT *Carotid intima-media thickness, *TWA LDL-C *Cumulative exposure time-weighted average of low-density lipoprotein cholesterol

### Logistic regression analysis of the influence of TWA LDL-C on IMT thickening

Logistic regression was used to analyze the effects of different TWA groups on IMT thickening in people 18–45 years old. The thickening of IMT (0 = no, 1 = yes) was used as the dependent variable, while the TWA LDL-C group was used as the independent variable. Model 1 was a single factor analysis, and model 2 corrected for age and sex. Model 3 adjusted for education level, body mass index, smoking, alcohol consumption, diabetes, hypertension, HDL-C, triglyceride and Hs-CRP on the basis of model 2. Model 4 added further adjustments for the use of adjustment of taking antihypertensive drugs and lipid-lowering drugs to model 3. Following the adjustments for possible influencing factors, the results showed that high TWA LDL-C was a risk factor for IMT thickening (*P* < 0.05), with *OR* values of 1.812(1.027 ~ 3.200) in the T3 group (Table 4).

**Table 4:** Logistic regression analysis of the effect of TWA LDL-C on IMT thickening

	B-value	SE	Wald	*P*-value	*OR* (95%*CI*)
Model 1					
T1			13.829	0.001	1.000
T2	0.528	0.266	3.939	0.047	1.696(1.007 ~ 2.856)
T3	0.921	0.251	13.468	< 0.001	2.513(1.536 ~ 4.111)
Model 2					
T1			2.929	0.231	1.000
T2	0.148	0.272	0.297	0.585	1.160(0.681 ~ 1.977)
T3	0.406	0.258	2.471	0.116	1.501(0.905 ~ 2.489)
Model 3					
T1			4.240	0.100	1.000
T2	0.389	0.302	1.662	0.197	1.475(0.817 ~ 2.664)
T3	0.592	0.289	4.185	0.041	1.807(1.025 ~ 3.184)
Model 4					
T1			2.242	0.120	1.000
T2	0.402	0.303	1.761	0.184	1.494(0.826 ~ 2.704)
T3	0.595	0.290	4.206	0.040	1.812(1.027 ~ 3.200)

Model 1: With IMT thickening (0 = no, 1 = yes) as the dependent variable and TWA LDL-C tertiles (with the first tertiles as the control group) as the independent variable, univariate Logistic regression analysis was performed. Model 2: Age and sex were corrected based on model 1. Model 3: On the basis of model 2, adjustments were added for education level, body mass index, smoking, alcohol consumption, diabetes, hypertension, HDL cholesterol, triglycerides, and C-reactive protein; Model 4: On the basis of model 3, the adjustment of taking antihypertensive drugs and lipid-lowering drugs was added

*TWA LDL-C *Cumulative exposure time-weighted average of low-density lipoprotein cholesterol, *IMT *Carotid intima-media thickness

### Sensitivity analysis of the effect of TWA LDL-C on IMT thickening

The thickening of IMT (0 = no, 1 = yes) was used as the dependent variable, and the TWA LDL-C group was used as the independent variable. The data of the patients who took antihypertensive drugs and lipid-lowering drugs (206 cases) were excluded. After adjusting for age, sex, education level, body mass index, smoking, drinking, diabetes, hypertension, HDL-C, triglyceride, and Hs-CRP. The results showed that the T3 group, with the highest TWA LDL-C, the *O*R value for IMT thickening is 1.850(0.988–3.464) (*P* = 0.055). The *P* for trend is 0.043 (Table 5).

**Table 5:** Sensitivity analysis of TWA LDL-C on IMT increase (*n* = 3445)

	B-value	SE	Wald	*P*-value	*OR* (95%*CI*)	*P* for trend
T1			4.065	0.131	1.000	0.043
T2	0.323	0.335	0.932	0.334	1.382(0.717–2.664)	
T3	0.615	0.320	3.693	0.055	1.850(0.988–3.464)	

Model: The group taking antihypertensive and lipid-lowering drugs was deleted, and the thickening of IMT (0 = no, 1 = yes) was used as the dependent variable, and the TWA LDL-C tertiles (the first tertiles was used as the control group) was used as the independent variable, adjusting for age, sex, education level, body mass index, smoking, alcohol consumption, diabetes, hypertension, HDL cholesterol, triglycerides, and C-reactive protein

*TWA LDL-C *Cumulative exposure time-weighted average of low-density lipoprotein cholesterol, *IMT *Carotid intima-media thickness

## Discussion

Our main discovery is that TWA LDL-C exhibits a positive dose–response relationship with IMT in young adults and serves as a risk factor for IMT thickening. The positive correlation between TWA LDL-C and IMT remains significant even when considering traditional risk factors. Moreover, the impact of antihypertensive and lipid-lowering drugs can mitigate the positive association between TWA LDL-C and IMT. Linear regression showed that TWA LDL-C was positively correlated with carotid IMT, with an increase of 1 mmol/L * year, and carotid IMT increased by 0.017 mm. As a categorical variable, high TWA LDL-C was a risk factor for carotid intima-media thickening, with the risk of carotid intima-media thickening in the T3 group being 1.812 times higher than that in the T1 group. It was further confirmed that TWA LDL-C was a risk factor for carotid intima-media thickening. In addition, the risk of IMT thickening in the T3 group excluding antihypertensive and lipid-lowering drugs increased by 4.3% compared with the main model, suggesting that antihypertensive and lipid-lowering drugs can reduce the effect of ATW LDL-C-induced IMT thickening. Therefore, early intervention is needed for individuals with high TWA LDL-C, and if necessary, antihypertensive and lipid-lowering drugs should be taken to reduce the risk of IMT thickening and prevent the occurrence of ASCVD in the later stage.

The previous studies have proved that LDL-C is an important risk factor for arterial intima-media thickening in different life cycles and ethnic groups [4, 12–15]. These studies were limited to a single measurement, reflecting only a short-term situation and potentially biased, thus overlooking long-term effects. The effects of accumulated hypertension and glucose and lipid metabolism disorders on adverse outcomes are stronger than those of single measurements [16, 17].

LDL-C is the primary intervention target for the prevention and treatment of ASCVD. The guidelines recommend that the ideal level of LDL-C in low-risk population in China should be < 2.6 mmol/L, and the marginal elevation should be ≥ 3.4 mmol/L and < 4.1 mmol/L [18]. Our study found that the risk of IMT thickening increased in the group with an elevated mean LDL-C margin in young adults ≤ 45 years of age. Therefore, we suggest that LDL-C should be maintained at an ideal level in young people. Awareness, treatment, and control of dyslipidemia in young people should be improved, and the elevated LDL-C level should be prevented or reduced through healthy lifestyle or the use of statins when necessary. This can reduce the risk of IMT thickening and prevent ASCVD, improving the overall health of the population and reducing the burden on society. The findings suggest that level of LDL-C is useful for risk stratification, and may inform strategies for early intervention in order to reduce the lifetime risk of developing atherosclerotic ASCVD. To prevent ASCVD, in addition to paying attention to blood lipids, other blood routine test indexes are also worth studying, such as platelet indices, especially Plateletcrit (PCT), mean platelet volume (MPV), platelet distribution width (PDW), platelet counts (PC) as markers for atherosclerotic cardiovascular disease (ASCVD), which is worth further studying in the future [19].

There are some limitations to this study. First, the average number of LDL-C measurements was 4 times, a relatively low number, and the numbers of acquisition varied. Second, the average cumulative exposure time was 6.3 years, and the follow-up time was relatively short. Third, there is a lack of data during childhood and adolescence to assess whether cumulative LDL-C exposure has an effect on younger age groups.Last,The subjects of this study were young people under 45 years old, so the proportion of carotid intima-media thickening was less. However, despite these limitations, the sample size of this study is large, and the weighted average value of cumulative exposure time to reflect cumulative exposure enhances the significance of the results as valuable reference.

## Conclusions

In summary, the current study reveal that TWA LDL-C during young adulthood were associated with carotid intima-media thickening as the risk factor of the incident of ASCVD. These findings suggest that past levels of LDL-C may inform strategies for primary prevention of ASCVD and that maintaining optimal LDL-C levels at an earlier age to prevent ASCVD.

## Data Availability

The datasets used during the current study are available from the corresponding author on reasonable request.
